# Prevalence of Non-Communicable Diseases and Healthcare Utilization Among Older Adults in Mongolia

**DOI:** 10.1093/geroni/igaf122.1907

**Published:** 2025-12-31

**Authors:** Zoljargalan Gantumur, Yifan Lou

**Affiliations:** Virginia Commonwealth University, Richmond, Virginia, United States; Virginia Commonwealth University, Richmond, Virginia, United States

## Abstract

Non-communicable diseases (NCDs) pose significant health burdens in Mongolia, a lower-middle-income landlocked country in Central Asia, where 16% of the population is projected to be of retirement age by 2045. NCDs are the leading causes of morbidity, mortality, and disability in later life, yet NCD-related research in Mongolia remains limited. Using nationally representative data, we investigated the prevalence, predictors, and health service utilization outcomes among older Mongolians. Data were from 1,989 adults aged 50+ in Mongolia’s National NCD Risk Factor Surveillance Survey (W4; 2019). NCDs were measured by the diagnosis of hypertension, raised cholesterol, cardiovascular diseases, and diabetes. We also consider the number of NCDs (0, 1, 2+). Logistic and multinomial logistic regression were used to understand the demographic and socioeconomic status (SES) predictors of NCDs and their relationships with healthcare utilization (doctor visits, outpatient hospital visits, and hospitalization). 64% of sampled older Mongolians had an NCD, with hypertension being the most prevalent. Nearly 49% received their diagnosis within the past year, and 24.1% had 2+ NCDs. Females are more likely to have 2+ NCDs. Employment status is also a strong predictor of having comorbidity. Socio-demographic predictors for each NCD vary, with low SES being the strongest predictor for cardiovascular diseases, but not for hypertension. Having 2+ NCDs increased all types of healthcare utilization compared to having none or one NCD, with each type of NCD driving greater use of different healthcare services. The findings inform targeted NCD management programs in Mongolia, particularly for women with multiple diagnoses.

